# Implementation of Single High-dose Liposomal Amphotericin B Based Induction Therapy for Treatment of HIV-associated Cryptococcal Meningitis in Uganda: A Comparative Prospective Cohort Study

**DOI:** 10.1093/cid/ciae413

**Published:** 2024-08-24

**Authors:** Jane Gakuru, Enock Kagimu, Biyue Dai, Samuel Okurut, Laura Nsangi, Nathan C Bahr, Michael Okirwoth, Olivie C Namuju, Joseph N Jarvis, David S Lawrence, Cynthia Ahimbisibwe, Jayne Ellis, Kizza Kandole Tadeo, David R Boulware, David B Meya, Lillian Tugume

**Affiliations:** Infectious Diseases Institute, Makerere University, Kampala, Uganda; Infectious Diseases Institute, Makerere University, Kampala, Uganda; Division of Biostatistics and Health Data Science, University of Minnesota, Minneapolis, Minnesota, USA; Infectious Diseases Institute, Makerere University, Kampala, Uganda; Infectious Diseases Institute, Makerere University, Kampala, Uganda; Division of Infectious Diseases & International Medicine, Department of Medicine, University of Minnesota, Minneapolis, Minnesota, USA; Infectious Diseases Institute, Makerere University, Kampala, Uganda; Infectious Diseases Institute, Makerere University, Kampala, Uganda; Clinical Research Department, London School of Hygiene and Tropical Medicine, London, United Kingdom; Botswana Harvard Health Partnership, Gaborone, Botswana; Clinical Research Department, London School of Hygiene and Tropical Medicine, London, United Kingdom; Botswana Harvard Health Partnership, Gaborone, Botswana; School of Pathology, Faculty of Health Sciences, University of the Witwatersrand, Johannesburg, South Africa; Infectious Diseases Institute, Makerere University, Kampala, Uganda; Infectious Diseases Institute, Makerere University, Kampala, Uganda; Clinical Research Department, London School of Hygiene and Tropical Medicine, London, United Kingdom; Infectious Diseases Institute, Makerere University, Kampala, Uganda; Division of Infectious Diseases & International Medicine, Department of Medicine, University of Minnesota, Minneapolis, Minnesota, USA; Infectious Diseases Institute, Makerere University, Kampala, Uganda; Division of Infectious Diseases & International Medicine, Department of Medicine, University of Minnesota, Minneapolis, Minnesota, USA; College of Health Sciences, Makerere University, Kampala, Uganda; Infectious Diseases Institute, Makerere University, Kampala, Uganda

**Keywords:** cryptococcal meningitis, HIV, liposomal amphotericin B, implementation, advanced HIV disease

## Abstract

**Background:**

In 2022, the World Health Organization (WHO) recommended a single 10 mg/kg dose of liposomal amphotericin B in combination with 14 days of flucytosine and fluconazole (AMBITION-cm regimen) for induction therapy of human immunodeficiency virus (HIV)-associated cryptococcal meningitis, based on the results of the multisite AMBITION-cm trial. We evaluated outcomes after real-world implementation of this novel regimen in Uganda.

**Methods:**

We enrolled Ugandan adults with cryptococcal meningitis into an observational cohort receiving the AMBITION-cm regimen with therapeutic lumbar punctures in routine care during 2022–2023. We compared 10-week survival and CSF early fungicidal activity with the outcomes observed in the AMBITION-cm clinical trial conducted at the same sites.

**Results:**

During 2022–2023, 179 adults were treated with the AMBITION-cm regimen via routine care and compared to the 171 adults randomized to the AMBITION-cm trial interventional arm in Uganda from 2018 to 2021. No significant difference in 10-week survival occurred between the observational cohort (68.6%; 95% confidence interval [CI]: 61.6%–76.3%) and AMBITION-cm trial participants in the intervention arm (71.7%; 95% CI: 65.2%–78.8%; absolute risk difference = −3.1%; 95% CI: −13.1% to 6.9%; *P* = .61). Early fungicidal activity did not differ (0.42 vs 0.39 log_10_CFU/mL/day; *P* = .80) between groups. Among observational cohort participants discharged alive initially and for whom follow-up data were available, the incidence of re-hospitalizations due to persistently elevated intracranial pressure was 2.8% (4/144).

**Conclusions:**

The AMBITION-cm regimen for cryptococcal meningitis resulted in similar outcomes as observed in the AMBITION-cm clinical trial when implemented in routine care. Intracranial pressure management during hospitalization and awareness after discharge are key components of optimizing outcomes.

## Teaser

In real world implementation, we found 10-week survival after cryptococcal meningitis using a single dose 10 mg/kg liposomal amphotericin B-based induction regimen did not differ from the outcomes observed in the AMBITION-cm randomized clinical trial.

## Graphical Abstract

**Graphical Abstract:**
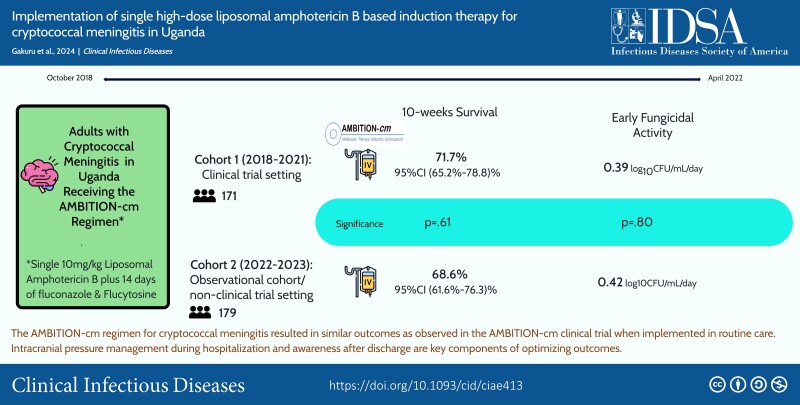
This graphical abstract is also available at Tidbit: https://tidbitapp.io/tidbits/implementation-of-single-high-dose-liposomal-amphotericin-b-based-induction-therapy-in-routine-care-for-treatment-of-hiv-associated-cryptococcal-meningitis-in-uganda-a-comparative-prospective-cohort-stud-b692ee8c-4574-4651-9bcf-7bf8c8d3afde?utm_campaign=tidbitlinkshare&utm_source=IO

Cryptococcal meningitis is the most common cause of human immunodeficiency virus (HIV)-associated meningitis accounting for ∼19% of AIDS-related deaths globally [1]. Historically, cryptococcal meningitis treatment consists of up to 14 days of intravenous amphotericin B. Prolonged amphotericin is associated with severe toxicities including anemia, hypokalemia, and nephrotoxicity [2]. Mortality in routine care in low-income countries is >50% with amphotericin B deoxycholate induction therapy [3, 4]. Furthermore, implementation challenges associated with long intravenous amphotericin regimens include: limited supply of antifungals, nosocomial infections, and the need for meticulous management of electrolyte abnormalities associated with amphotericin [2]. Therefore, shorter induction regimens have been investigated.

The AMBITION-cm trial was a phase III, randomized clinical trial that demonstrated non-inferiority for 10-week survival of a single 10 mg/kg dose of liposomal amphotericin B combined with 14-days of flucytosine and fluconazole (AMBITION-cm regimen) as compared to the standard of care amphotericin B deoxycholate at 1 mg/kg/day plus flucytosine for 7-days, followed by fluconazole 1200 mg/day for 7-days [5]. This AMBITION-cm regimen also demonstrated improved safety [5]. Subsequent analyses showed that the AMBITION-cm regimen was cost effective and acceptable in low- and middle-income settings [6, 7].

The World Health Organization (WHO) 2022 cryptococcal guidelines recommend the AMBITION-cm regimen as the preferred therapy [8]. We sought to describe the outcomes after implementing the AMBITION-cm regimen in routine care and compare outcomes as observed in the AMBITION-cm clinical trial.

## METHODS

### Study Design

This is a comparative study of 2 prospective cohorts of persons with HIV and cryptococcal meningitis. We describe the outcomes of participants treated with the AMBITION-cm regimen after national implementation compared to the outcomes observed in the clinical trial. Uganda was the largest recruiting country for the AMBITION-cm trial. Between October 2018 and February 2021, 342 participants with cryptococcal meningitis were enrolled from Mbarara Regional Referral Hospital and Kiruddu National Referral Hospital, of whom 171 adults were randomized to the AMBITION-cm trial intervention arm. Following WHO guidelines revision in April 2022 [8], we subsequently enrolled an observational cohort treated with the AMBITION-cm regimen as part of routine care at both trial sites and at a re-opened Mulago National Specialized Hospital. The analyses reported herein represent participants enrolled from 13 September 2022 to 23 October 2023.

Participants or their surrogates provided written informed consent. The Mulago Hospital Research and Ethics Committee (MHREC 1246) and the Uganda National Council of Science and Technology approved the studies. The University of Minnesota institutional review board approved the observational cohort.

### AMBITION-cm Trial Participants

AMBITION-cm trial participants were adults (≥18 years*)* with first-episode cryptococcal meningitis, diagnosed by cerebrospinal fluid (CSF) cryptococcal antigen (CrAg) lateral flow assay (Immy, Norman, Oklahoma, USA). Individuals were excluded who were pregnant, currently breastfeeding, unable to attend follow-up visits, received >2 days of prior antifungal therapy, or had laboratory exclusion criteria of: neutrophil count <50 000/μL, platelets <50 000/μL and, alanine aminotransferase >5-times normal [5]. To enable rapid treatment of critically ill participants, the AMBITION-cm trial provided for late exclusion/early withdrawal based on laboratory exclusion criteria [9]. Participants withdrawn for late exclusion criteria were followed for outcomes.

### Observational Cohort

The observational prospective cohort consisted of adults with HIV aged ≥18 years, with cryptococcal meningitis diagnosed by CSF CrAg lateral flow assay. Relapse of cryptococcal meningitis was diagnosed using CSF fungal culture only [10]. Only pregnant or breastfeeding women and those with paradoxical immune reconstitution inflammatory syndrome were excluded. Participants within the comparative cohort received a single 10 mg/kg dose liposomal amphotericin B, plus 14-days of flucytosine 100 mg/kg/day and fluconazole 1200 mg/day for induction [8]. These antifungals were provided through the Uganda Advanced HIV Disease program with support from Unitaid and Clinton Health Access Initiative [11]. Thereafter, the continuation phase therapy was fluconazole 800 mg/day through 10 weeks, then 200 mg/day for secondary prophylaxis.

### Clinical Care

All AMBITION-cm trial participants received ancillary care as stipulated in the protocol, including a minimum of 3 lumbar punctures on days 1, 7, and 14 and laboratory tests as follows: urea, creatinine and electrolytes on days 1, 3, 5, 7, 10, 12, 14, and 28, and complete blood count and alanine aminotransferase on days 1, 7, 14, and 28 [9]. Conversely, participants in the observational cohort had a diagnostic lumbar puncture and early repeat lumbar puncture (target of day 3 ± 1) with measurement of CSF opening pressure using imported manometers. Additional therapeutic lumbar punctures performed at physician discretion on days 7, 14, or with ongoing symptoms. Follow-up laboratory monitoring was performed only at physician discretion, based on baseline abnormalities with the knowledge of the known safety profile of AMBITION-cm regimen. The study team or hospital staff prescribed potassium chloride and magnesium chloride tablets for three days as standardized electrolyte supplementation.

All participants had daily clinical reviews during hospitalization. For AMBITION-cm trial participants, the duration of hospitalization was a minimum of 7 days from enrollment. Conversely, in the comparison cohort, participants were discharged according to physician discretion from day 5 onward. Following hospital discharge, AMBITION-cm participants were reviewed every 2 weeks as outpatients until 10-weeks post diagnosis as per protocol. Clinical reviews after discharge for participants within the observational cohort, were performed as needed with antiretroviral therapy (ART) initiation targeted for 4–6 weeks and a review at 12-weeks post-diagnosis to assess neurocognitive status.

### Statistical Methods

Baseline demographics and clinical characteristics were summarized overall and by cohort using medians with interquartile range or percentages. We compared baseline characteristics using Wilcoxon rank sum test for continuous variables, Pearson's chi-squared test for categorical variables with expected cell counts ≥5 and Fisher's exact for categorical variables with expected cell counts <5. Mortality at 10 weeks was estimated using the Kaplan-Meier estimators with log rank testing. We reported unadjusted hazard ratios estimated via univariate Cox proportional hazard model. To account for confounding and imbalances in the baseline characteristics between groups, adjusted hazard ratios were estimated using multivariate Cox proportional hazard models, where the baseline covariates were selected via LASSO regression. Participants terminated early due to late withdrawal criteria in AMBITION-cm trial were all included in this analysis.

Due to the differences in follow-up between the AMBITION-cm trial and the observational cohort, we conducted sensitivity analysis for the mortality outcomes using the inverse-probability-of-censoring weighted Kaplan-Meier estimates. In the presence of missing data, complete cases were used for univariate analysis, and mean imputations were used for multivariate analysis.

For participants with positive CSF cultures who had at least 1 follow-up quantitative culture measurement during the initial hospitalization, early fungal activity (EFA), which measures the rate of cryptococcal clearance from CSF, was calculated as the slope of a simple linear regression between log_10_-transformed fungal quantitative culture CFU/mL and days from initiation of antifungal therapy. We compared EFA between groups as a continuous variable.

As the lab monitoring in the observational cohort was abbreviated, a direct comparison was not made with the incidence of lab adverse events discovered with intense safety monitoring in a clinical trial.

## RESULTS

First, during 2018–2021, 342 adults with cryptococcal meningitis were recruited as part of the AMBITION-cm clinical trial in Uganda, among whom 171 were randomized to receive single 10 mg/kg dose of liposomal amphotericin B combined with 14 days of flucytosine and fluconazole. Eight participants were terminated early due to meeting late exclusion criteria in the Ambition-CM trial. All trial participants were followed up until death or 10 weeks by the study team except 2 of the 8 participants who were terminated early in AMBITION-cm trial due to late exclusion criteria. Second, from September 2022 to October 2023, 179 adults with cryptococcal meningitis were recruited into the observational cohort and treated with the same induction regimen in a non-clinical trial setting, not following an exact clinical trial protocol. Outcome data to 10 weeks were available for 144 of these 179 participants with 34 (19%) lost to follow-up, at a median of 13 days (range: 4–42) from admission. Vital status at the time of discharge was known for all 34 participants lost to follow up. The overall median age was 36 years (interquartile range [IQR]: 30–42 years), and the majority (60%) were male (Table 1). At diagnosis, most of the participants were ART-naive (59%), and 34% had altered mental status with Glasgow coma scale score <15.

**Table 1.** Baseline Characteristics of the Two Study Populations With HIV and Cryptococcal Meningitis in Uganda

Characteristic	Observational Cohort N = 179	AMBITION-cm Trial N = 171	*P* Value
Age, y	37 (30, 42)	36 (30, 42)	.881
Biological sex, female	71 (40%)	70 (41%)	.809
Cryptococcal screening referral	85 (47%)	56 (35%)	.020
Antiretroviral therapy status	…	…	.002
ART-Naive	121 (68%)	85 (50%)	
Currently receiving ART	42 (23%)	67 (39%)	
Prior ART experienced	16 (8.9%)	19 (11%)	
Time on ART, wks	14 (4, 25)	12 (2, 70)	.760
Glasgow coma scale score <15	66 (37%)	52 (30%)	.201
Hemoglobin, g/dL	11.6 (9.7, 13.4)	11.5 (9.7, 13.0)	.385
CD4 cells/µL	32 (15, 80)	20 (8, 50)	.001
CSF opening pressure, mm H_2_O	240 (150, 326)	215 (140, 300)	.243
CSF opening pressure >200 mm H_2_O	93 (57%)	91 (54%)	.559
CSF protein, mg/dL	60 (40, 125)	80 (40, 119)	.666
CSF white cells/µL	5 (4, 110)	<5 (4, 46)	.019
Sterile baseline CSF culture	40 (24%)	24 (15%)	.034
CSF *Cryptococcus* log_10_ CFU/mL	4.63 (3.74, 5.06)	4.88 (3.54, 5.56)	.096

Values are median (IQR) or n (%).

*P* values via calculated via Wilcoxon rank-sum test or Pearson χ^2^ test; Fisher exact test.

Abbreviations: AMBITION-cm, single 10 mg/kg dose of liposomal amphotericin B combined with 14-days of flucytosine and fluconazole; ART, antiretroviral therapy; CFU, colony forming units; CrAg, Cryptococcal antigen; CSF, cerebrospinal fluid; HIV, human immunodeficiency virus.

Baseline differences existed between the two study cohorts with the roll out of CrAg screening in Uganda. The observational cohort had a higher proportion of ART-naive participants compared with the AMBITION-cm trial group (68% vs 50%, *P* < .001). The proportion of individuals with sterile CSF fungal culture at meningitis diagnosis was higher in the observational cohort compared to the clinical trial group (24% vs 15%, *P* = .034). Among participants with non-sterile CSF cultures, initial fungal burden was lower in the observational cohort than in the clinical trial group with a median CSF quantitative cryptococcal culture of 43 000 (IQR: 5500 to 15 500) CFU/mL versus 76 500 (IQR: 3500 to 360 000) CFU/mL (*P* = .096). The observational cohort enrolled 3.6% (6/167) with culture-positive relapse while the AMBITION-cm trial only enrolled those with a first episode. In addition, there was a higher proportion of participants referred via the public health facility CrAg screening program in the observational cohort compared to the clinical trial group (47% vs 35%, *P* = .020).

**Figure 1.**
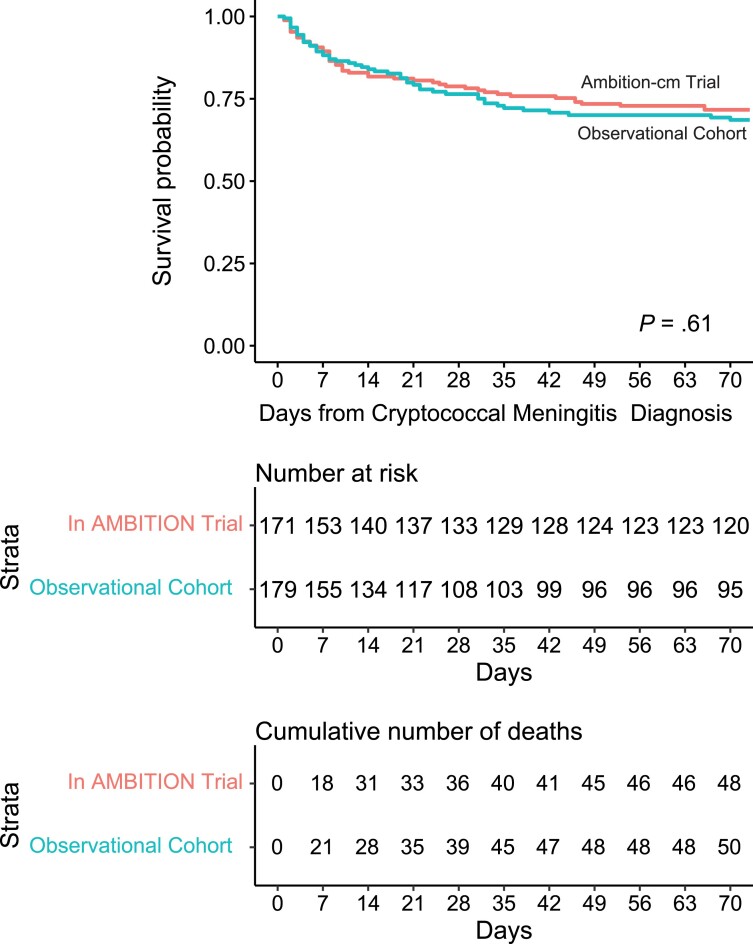
Cumulative 10-week all-cause mortality after cryptococcal meningitis. Comparison between the observational cohort in routine care and clinical trial group (AMBITION-cm trial intervention arm). Survival through 10 weeks did not statistically differ across study groups. Outcome data to 10 wks were available for 81% (145/179) of observational cohort participants with 32 in total lost to follow-up by 4 wks, and 34 (17%) in total lost to follow-up by 10 wks after diagnosis with right-hand censoring. One participant died on day 70. Abbreviation: AMBITION-cm, single 10 mg/kg dose of liposomal amphotericin B combined with 14-days of flucytosine and fluconazole.

The primary outcome was 10-week survival. Among the 171 AMBITION-cm intervention arm participants, the 10-week survival probability using the Kaplan-Meier estimator was 71.7% (95% confidence interval [CI]: 65.2%–78.8%), whereas among the 179 participants from the observational cohort, the 10-week survival probability using the Kaplan-Meier estimator was 68.6% (95% CI: 61.6%–76.3%) (Figure 1). No statistically significant difference in the 10-week survival occurred with an absolute risk difference of −3.1%, (95% CI: −13.1% to 6.9%; log-rank *P* = .61) in the observational cohort compared with AMBITION-cm trial cohort. In the unadjusted analyses, 10-week survival did not statistically differ in the observational cohort (unadjusted hazard ratio = 1.11; 95% CI: .75, 1.65, *P* = .61) as compared to the AMBITION-cm clinical trial cohort. When adjusting for baseline differences in demographics, the 10-week survival did not differ between groups (adjusted hazard ratio = 1.17; 95% CI: .74–1.86; *P* = .51) after adjusting for age, altered mental status, ART status, fungal burden, CSF pleocytosis with ≥5 white cells/μL, and anemia (Table 2).

**Table 2.** Ten-week Survival Among Participants With HIV and Cryptococcal Meningitis Who Received the AMBITION-cm Regimen in the Observational Cohort Versus in the Clinical Trial

	Crude Hazard Ratio (95% CI)	*P V*alue	^a^Adjusted Hazard Ratio (95% CI)	*P V*alue
Observational cohort versus AMBITION-cm clinical trial cohort	1.11 (.75, 1.65)	.613	1.17 (.74, 1.86)	.510
Age, per 10 y	1.28 (1.06, 1.55)	.010	1.34 (1.10, 1.63)	.004
Biological sex, female	0.95 (.63, 1.42)	.801		…
ART status				…
ART naïve	(Reference group)		…	…
Currently on ART	1.10 (.72, 1.69)	.662	…	…
Prior ART experienced	1.66 (.30, 1.44)	.298	…	…
Duration of ART				
ART ≤ 2 weeks	(Reference group)		…	…
ART > 2 weeks	1.03 (.45, 2.36)	.952	1.13 (.45, 2.82)	.792
Not on ART	0.86 (.40, 1.88)	.713	0.74 (.31, 1.75)	.488
Glasgow coma scale < 15	2.54 (1.71, 3.78)	<.001	2.43 (1.56, 3.79)	<.001
CD4 per 10 cell/µL	0.98 (.95, 1.01)	.252	…	…
Hemoglobin, g/dL	0.90 (.82, .99)	.028	0.88 (.80, .96)	.006
CSF opening pressure, per 10 mmH_2_O	1.01 (.99, 1.02)	.466	…	…
CSF protein, per 10 mg/dL	0.97 (.94, 1.00)	.076	…	…
CSF white cells, per 10 cells/µL	0.95 (.92, .98)	.003	0.95 (.91, .99)	.009
Sterile diagnostic culture	0.60 (.33, 1.11)	.103	…	…
CSF culture per 1 log_10_ CFU/mL	1.20 (1.01, 1.43)	.037	1.13 (1.01, 1.26)	.034
CrAg screening program referrals	0.78 (.52, 1.18)	.244	…	…

Abbreviations: AMBITION-cm, single 10 mg/kg dose of liposomal amphotericin B combined with 14-days of flucytosine and fluconazole; ART, antiretroviral therapy; CI, confidence interval; CrAg, cryptococcal antigen; CSF, cerebrospinal fluid; CFU, colony forming units; HIV, human immunodeficiency virus.

^a^Adjusted for baseline age, weeks on ART, Glasgow coma scale, hemoglobin, CSF white cell count, and quantitative CSF culture.

In the sensitivity analysis to partially account for the loss to follow-up in the observational arm, the inverse-probability-of-censoring weighted Kaplan-Meier estimates demonstrated similar survival in the observational cohort and the clinical trial group (Supplementary Figure 1), consistent with the primary results.

### Secondary Outcomes

Among the 171 AMBITION-cm trial participants, 2-week survival was 81.7% (95% CI: 76.1%–87.8%) versus 84.0% (95% CI: 78.7%–89.6%) in the observational cohort as estimated by Kaplan-Meier function (log rank *P* = .60). In both unadjusted, and adjusted survival analyses, 2-week survival was similar across groups (Supplementary Table 1).

The mean rate of CSF cryptococcal clearance was similar with mean early fungicidal activity of 0.42 (95% CI: .31 to .53) log_10_CFU per mL CSF per day in the observational cohort and 0.39 (95% CI: .34 to .45) log_10_ CFU/mL/day in the AMBITION-cm trial group (*P* = .80).

**Figure 2.**
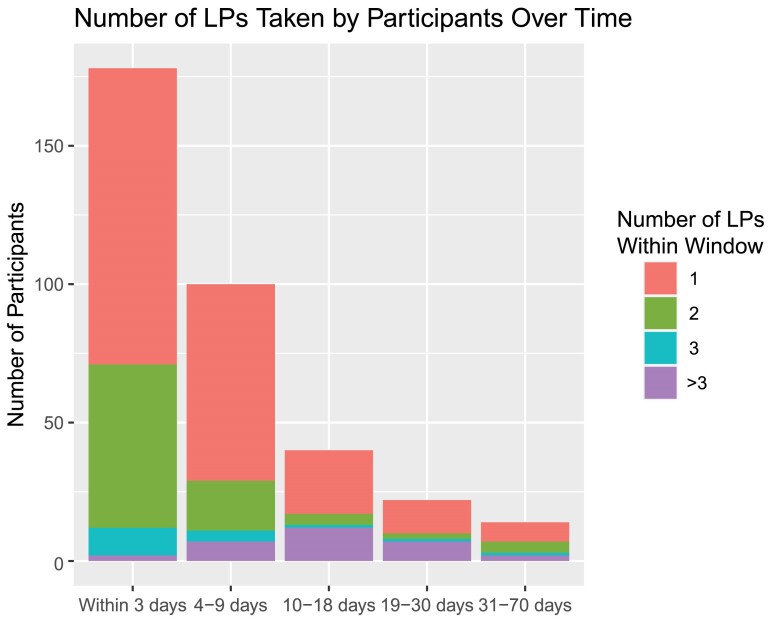
Frequency of lumbar punctures over time among participants in the observational cohort. The number of days from diagnosis of cryptococcal meningitis were categorized as follows: Day 1 window (within 3 d), day 7 window (4–9 d), and day 14 window (10–18 d); ≥2 d, over time. Number of days from diagnosis of cryptococcal meningitis. Abbreviation: LP, lumbar puncture.

The median duration of hospitalization was 11 days (IQR 7–14) in the observational cohort. Twenty percent were hospitalized for <7 days, 59% for 7 to 14 days, and 21% for >14 days. During the 14 days of induction therapy, 52% (93/179) of the participants had at least 1 follow-up laboratory monitoring test, and 4% (4/93) of participants with any follow-up testing developed severe (grade 3) or life-threatening (Grade 4) laboratory adverse events (Table 3). The median number of lumbar punctures (LP) was 2 (IQR 1–3) All participants had at least 1 LP, 74% had at least 2 LPs, and 43.5% had more than 2 LPs (Figure 2).

**Table 3.** New or Worsening Lab Abnormalities in the Observational Cohort, Among 93 Patients With at Least 1 Follow-up lab Measurement

AE Severity Grade^a^	Number of Participants (n = 93)
Creatinine	
3	0 (0%)
4	0 (0%)
Hemoglobin	
3	1 (1.1%)
4	2 (2.2%)
Potassium	
3	1 (1.1%)
4	0 (0%)

Abbreviation: adverse event.

^a^The 2017 Division of AIDS (DAIDS) grading scale for severity of adverse events.

The incidence of rehospitalizations within 10 weeks of follow-up was 6.2% (9/144) among participants who were discharged alive, and for whom follow-up data were available. The incidence of rehospitalization with elevated intracranial pressure (>200 mm H_2_O) was 2.8% (4/144) within 10 weeks of follow-up.

## DISCUSSION

We demonstrated similar 10-week survival after cryptococcal meningitis when implementing the WHO 2022 treatment guidelines in routine care as per the AMBITION-cm trial regimen. We demonstrated that survival outcomes are comparable despite less rigorous laboratory monitoring in the real-world setting versus AMBITION-cm trial. The WHO guidelines include a basic package of ancillary care including control of intracranial pressure, which remains critical to optimize survival in addition to antifungal medicines [12, 13].

Within our observational cohort, 2-week mortality was 16%, and one fifth of participants were hospitalized for <7 days. These clinical outcomes are much improved compared to historical cryptococcal meningitis cohorts in routine care settings in Africa when the standard induction regimen was amphotericin B deoxycholate with fluconazole. For instance, in-hospital mortality was approximately 40% in rural Uganda from 2017 to 2019 [14]. The reasons for the marked improvement in cryptococcal meningitis survival in routine care settings after 2019 are multifactorial including: widespread implementation of CrAg screening programs [15], lifesaving access to flucytosine and liposomal amphotericin B through the Unitaid and the Clinton Health Access Initiative [16], and effective ancillary clinical care with the provision of manometers and electrolyte support [12, 13].

The 2022 WHO guidelines for cryptococcal disease recommend laboratory monitoring of serum potassium and creatinine on days one and three of induction therapy and hemoglobin on day 1 and day 7 [8]. The WHO guidelines additionally recommend an initial diagnostic lumbar puncture followed by a therapeutic lumbar puncture early during treatment irrespective of presence of symptoms [8]. During the observational cohort, three quarters of patients received ≥2 lumbar punctures including the initial diagnostic lumbar puncture; however, laboratory monitoring was performed at physician discretion. The majority of the patients had a documented baseline complete blood count, serum creatinine, and serum potassium, but only half had follow-up laboratory tests 3–10 days later. The limited laboratory monitoring conducted during this observational cohort was likely adequate when using the single 10 mg/kg dose liposomal amphotericin regimen. In fact, only 3% of the overall AMBITION-cm trial population developed severe anemia with hemoglobin <6.5 g/dL, 2% developed severe hypokalemia (<2.5 mmol/L), and none developed potentially life threatening hypokalemia (<2 mmol/L) within 21 days of initiating antifungals [5]. The resources that would otherwise be utilized for rigorous laboratory monitoring should be channeled to purchasing manometers, spinal needles, and local anesthetic for performing therapeutic lumbar punctures.

The single intravenous liposomal amphotericin B regimen is potentially cost saving due to the prospect of shorter hospital stay in comparison to longer courses of intravenous amphotericin [6]. The median hospital stay in this observational cohort was 11 days, yet historically, hospital stay of at least 14 days has been standard practice. The benefits of reduced hospital stay could potentially be offset by frequent rehospitalization and excess mortality after discharge [17–19]. However, the incidence of readmissions attributed to elevated intracranial pressure within 10 weeks of follow-up was ∼3% in the observational cohort. These findings imply that patients should be instructed about the possibility of persistent/recurrent elevated intracranial pressure after discharge and to seek care immediately. The theoretical concerns with using single-dose amphotericin that patients will be discharged prematurely with uncontrolled intracranial pressure did not manifest herein. Economic evaluation of interventions for monitoring recurrence of elevated intracranial pressure after early discharge, such as telephone reviews, are warranted. Telephone reviews after early hospital discharge could potentially reduce the cost associated with frequent in-person reviews after discharge and also identify patients that require in-person reviews for management of intracranial pressure, have failure to thrive, and/or require screening for other coinfections.

We acknowledge that the clinical trial group enrolled in 2018–2021 were a generally “sicker” population than those enrolled in 2022–2023 as evidenced by significantly higher baseline fungal burden and greater proportion of patients with fungal growth in CSF cultures. This change was related to nearly half of patients in the 2022–2023 cohort having been referred for hospital care through an established routine CrAg screening program. CrAg screening programs directly impact on the survival of patients with cryptococcosis by providing an opportunity for early diagnosis of central nervous system disease [15].

The main limitation of this study is that 19% of the observational cohort group were effectively lost to follow-up after hospital discharge, a limitation that is inherent of observational studies conducted in real world settings. Therefore, mortality beyond hospitalization and rehospitalization outcomes should be interpreted with caution. The observed survival rate through 10 weeks and the incidence of rehospitalization in the observational cohort could be an underestimated of the true value. For the primary outcome (10-week survival), we minimized the bias due to differences in follow-up in the clinical trial versus observational cohort by conducting sensitivity analyses; however, we cannot fully account for loss to follow-up by statistical means and may have underestimated post-discharge mortality in this group. Additionally, the hospitals at which the observational cohort was enrolled are not representative of most real-world settings in Africa. The observational cohort was enrolled at tertiary hospitals by a research team consisting of highly experienced research nurses who had been previously trained for the AMBITION-cm trial, indicating that the clinical expertise may not have been representative of a typical routine care setting. However, the medical doctors involved in the observational cohort were newly trained, having not participated in the AMBITION-cm trial. This underscores the importance of training health care workers, both nurses and physicians, in routine care settings for standardized cryptococcal care in accordance with best practices. Further data are needed from more health care facilities in low- and middle-income settings. In the absence of the availability of manometers (which are unavailable outside of research studies) and conducting therapeutic lumbar punctures [12, 13], survival would be anticipated to be ∼15% worse [13]. Instead of performing extensive follow up laboratory monitoring, which the observational cohort suggests may be unnecessary, health systems would achieve better survival by investing in manometers at a cost of US$10 each to enable measurement and control of intracranial pressure.

In conclusion, the effectiveness of the AMBITION-cm regimen in routine care settings in Africa may be comparable to the efficacy demonstrated in the clinical trial when implemented as part of a basic package of care including at least two routine lumbar punctures for management of intracranial pressure. Frequent laboratory monitoring may not be necessary as a routine for most patients treated with the single intravenous amphotericin regimen as per the WHO 2022 guidelines. Shorter hospital stays are possible with the single 10 mg/kg dose amphotericin regimen; however, we recommend a follow-up mechanism after early discharge to manage recurrence of elevated intracranial pressure.

## Supplementary Data

Supplementary materials are available at *Clinical Infectious Diseases* online. Consisting of data provided by the authors to benefit the reader, the posted materials are not copyedited and are the sole responsibility of the authors, so questions or comments should be addressed to the corresponding author.

## Supplementary Material

ciae413_Supplementary_Data

## Notes

***Financial support*.** This work was supported by the United States National Institute of Neurological Disorders and Stroke (grant numbers R01 NS086312, K23 NS110470), the Fogarty International Center (grant number D43 TW009345), and the National Institute of Allergy and Infectious Diseases (grant numbers R01 AI170158, K23 AI138851). Additional contributing support was received from the Wellcome Trust (grant number 203905/Z/16/Z) awarded to J. E. for a Clinical PhD Fellowship. J. N. J. and D. S. L. receive salary funding from the UK National Institute for Health and Care Research (NIHR) (grant numbers NIHR-RP-2017-08-ST2-012, NIHR134342) to support improving global health. The views expressed in this publication are those of the authors and not necessarily those of the NIHR or UK government. The AMBITION-cm trial was supported by a grant (number TRIA2015-1092) through the European and Developing Countries Clinical Trials Partnership (EDCTP), with assistance from the Swedish International Development Cooperation Agency, as well as by funding from the UK Department of Health and Social Care, the UK Foreign Commonwealth and Development Office, the UK Medical Research Council, and Wellcome Trust via the Joint Global Health Trials scheme (grant number MR/P006922/1).
