# Supplementary material for: Implementation of Single High-dose Liposomal Amphotericin B Based Induction Therapy for Treatment of HIV-associated Cryptococcal Meningitis in Uganda: A Comparative Prospective Cohort Study
Source: Clin Infect Dis. 2024 Aug 24;80(2):417–24. doi: 10.1093/cid/ciae413 (PMC11848271; doi:10.1093/cid/ciae413)
Supplement: ciae413_Supplementary_Data [file ciae413_supplementary_data.docx]

**Supplementary Table S1. Univariate and multivariable analyses of two-week survival among cryptococcal meningitis participants enrolled in the observational cohort vs AMBITION-cm clinical trial setting.**

| Characteristic | Hazard Ratio  (95% CI) | p-value | ^1^Adjusted HR  (95% CI) | p-value |
| --- | --- | --- | --- | --- |
| Observational cohort vs Ambition-cm trial cohort | 0.87 (0.52, 1.46) | .604 | 0.85 (0.46, 1.57) | .597 |
| Age, per 10 years | 1.19 (0.93, 1.52) | .175 | 1.20 (0.93, 1.55) | .155 |
| Time on ART |  |  |  |  |
| ART < 2 weeks |  |  |  |  |
| ART > 2 weeks | 1.62 (0.48, 5.50) | .439 | 1.54 (0.44, 5.35) | .496 |
| Not on ART | 1.17 (0.36, 3.78) | .796 | 0.81 ( 0.24, 2.73) | .735 |
| Glasgow Coma Scale score <15 | 3.36 (2.00, 5.65) | <.001 | 3.58 (1.98, 6.48) | <.001 |
| CD4 Counts, per 10 cells/µL | 0.98 (0.94, 1.02) | .388 |  |  |
| Hemoglobin, g/dL | 0.86 (0.77, 0.97) | .012 | 0.84 (0.75, 0.95) | .004 |
| CSF Protein, per 10 mg/dL | 0.96 (0.91, 1.00) | .070 |  |  |
| CSF white cells per 10 cells/μL | 0.96 (0.92, 1.00) | .046 | 0.96 (0.92, 1.00) | .076 |
| Sterile diagnostic cultures | 0.54 (0.24, 1.19) | .127 |  |  |
| CSF Culture per 1 log_10_ CFU/mL | 1.23 (0.98, 1.54) | .068 | 1.17 (1.01, 1.35) | .032 |

Abbreviations: ART = antiretroviral therapy; CrAg = cryptococcal antigen, CSF = cerebrospinal fluid; CFU = colony forming units.

^1^Adjusted for age, weeks on ART, Glasgow coma scale, hemoglobin, CSF white cell count, and CSF quantitative culture.

**Supplementary Figure S1: Inverse Probability of Censoring Weighted Kaplan Meier Curve for 10-week mortality.**


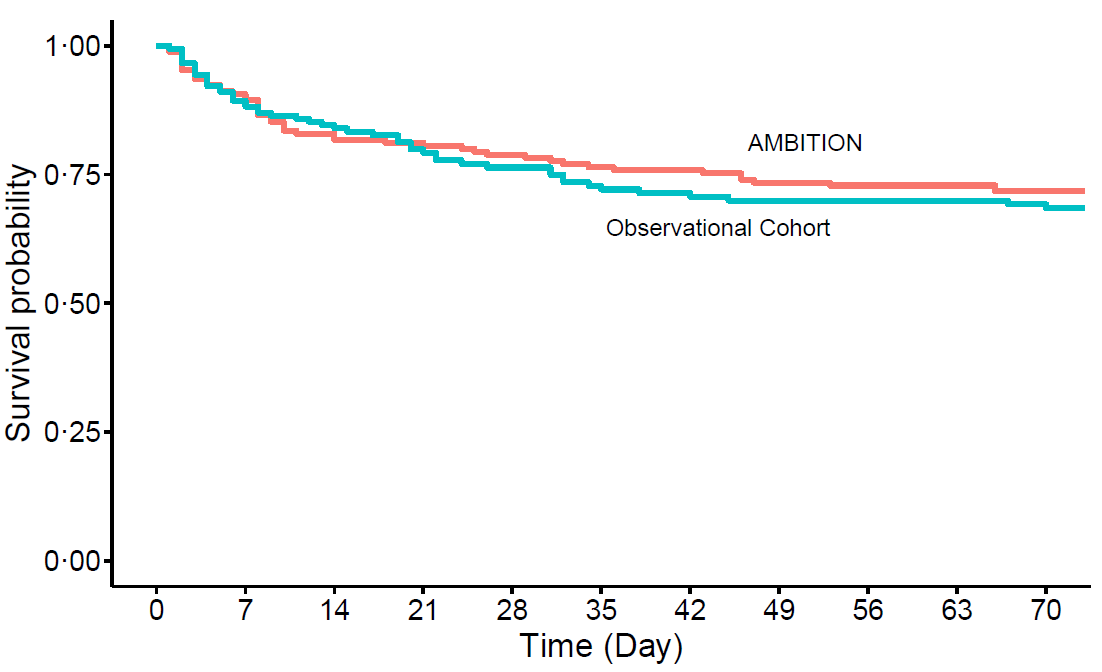


There was no statistically significant difference in 10-week mortality across the two groups after accounting for informative censoring using inverse probability weighting.
